# High-Precision Ultrasonic Anemometry System Based on Polyvinylidene Fluoride Piezoelectric Film and Variational Mode Decomposition-Extended Kalman Filter Joint Optimization

**DOI:** 10.3390/s26051482

**Published:** 2026-02-26

**Authors:** Haodong Niu, Yunbo Shi, Kuo Zhao, Jinzhou Liu, Qinglong Chen, Xiaohui Yang

**Affiliations:** 1 School of Measurement and Control Technology and Communication Engineering, Harbin University of Science and Technology, Harbin 150080, China; 2010600001@stu.hrbust.edu.cn (H.N.); zhaokuo_96@yeah.net (K.Z.); liujinzhou0709@outlook.com (J.L.); chenqinglong1008@yeah.net (Q.C.); 420610159@stu.hrbust.edu.cn (X.Y.); 2 China Higher Educational Key Laboratory for Measuring & Control Technology and Instrumentations of Heilongjiang Province, Harbin 150080, China

**Keywords:** ultrasonic anemometry, polyvinylidene fluoride, variational mode decomposition, extended Kalman filter

## Abstract

Ultrasonic wind speed measurements performed in complex flow fields face challenges related to low signal-to-noise ratio (SNR) and non-stationary waveform distortion. In this study, we aim to address this issue by proposing a measurement system that employs a polyvinylidene fluoride (PVDF) piezoelectric film ultrasonic transducer integrated with a microphone (MIC). In addition, a signal processing framework is proposed based on the joint optimization of variational mode decomposition (VMD) and an extended Kalman filter (EKF) and integrating cross-correlation interpolation. By leveraging the low Q-factor and wide bandwidth characteristics of the PVDF, the system achieved omnidirectional transmission and high-fidelity reception within a compact structural design. The experimental results demonstrated that the proposed VMD-reference signal-assisted EKF method enhanced the SNR by approximately 26% and reduced the wind speed measurement error by approximately 35% compared with the conventional EKF. The proposed system exhibited superior robustness and measurement linearity across a wide wind speed range of 0–60 m/s. The proposed scheme significantly enhances the accuracy and environmental adaptability of ultrasonic wind speed measurements and provides an essential theoretical basis and engineering reference for the development of precision instruments in fields such as meteorological monitoring and wind energy assessment.

## 1. Introduction

Accurate wind speed measurements are of critical importance in fields such as meteorological monitoring, aerospace, wind power generation, and engineering construction [1,2,3]. Currently, mainstream wind speed measurement methods include mechanical, hot-wire, Pitot tube, and ultrasonic techniques [4,5,6]. However, conventional mechanical anemometers suffer from inherent limitations such as wear on rotating components, response hysteresis, and starting wind speed thresholds. These defects limit the lifespan of the systems and hinder their ability to meet the demands of low wind speed measurements and rapid responses. Conversely, an ultrasonic wind measurement technology based on the propagation characteristics of sound waves in fluids offers distinct advantages, including the absence of mechanical inertia and wear, high sensitivity, and zero starting threshold wind speeds [7,8,9]. Existing hardware systems predominantly employ piezoelectric ceramic (PZT) transducers for ultrasound generation. Despite their high sensitivity, the high-quality factor (High-Q) of PZT materials results in prolonged ringing. Furthermore, their strong directivity imposes a heavy computational burden during subsequent signal processing and limits the structural flexibility of the measurement system [10,11].

The most commonly employed methods for ultrasonic fluid speed measurements include phase difference, cross-correlation, and threshold-based methods. Threshold detection is a standard technique used for identifying ultrasonic signals. In this approach, the signal arrival time is determined based on a predefined minimum voltage amplitude [12]. However, in high-wind-speed environments, the received signal typically exhibits a low signal-to-noise ratio (SNR). The noise interference may exceed the threshold level, making it challenging to define an appropriate voltage threshold. Furthermore, a single wind speed measurement requires the sequential driving of piezoelectric transducers to perform two independent forward and reverse ultrasonic time-of-flight (ToF) measurements. This sequential process introduces significant errors in environments with rapidly fluctuating wind speeds.

Regarding the processing of received ultrasonic signals, Liu et al. proposed a detection framework that combined the Hilbert–Huang transform (HHT) with high-order statistics (kurtosis). This method extracts intrinsic mode functions (IMFs) via empirical mode decomposition (EMD) and constructs a time–frequency spectrum using the Hilbert transform to preliminarily locate the first arrival time interval. Within this interval, a fourth-order kurtosis feature function is calculated using a sliding window, and the precise first arrival time is determined based on the local minimum. However, this method relies solely on the HHT to locate the arrival time range without explicit denoising. Although the subsequent kurtosis calculation can suppress Gaussian noise, it remains sensitive to non-stationary noise, potentially amplifying measurement errors [13].

For ultrasonic ToF estimation, dos Santos et al. proposed a fusion framework based on the extended Kalman filter (EKF) and zero-crossing detection (ZCD) [14]. Compared with the study by Angrisani et al., which relied solely on the EKF for signal processing [15], this method utilized the reconstructed signal output from the EKF and identified the first zero-crossing point as the ToF reference via ZCD. This approach effectively circumvents the parameter coupling errors in the EKF arising from envelope distortion. However, the accuracy of ToF estimation using ZCD is heavily dependent on the system sampling rate (*fs*). Furthermore, as the wind speed increases, the resulting distortion of the echo signal can lead to significant measurement errors.

This study addresses the aforementioned hardware and algorithm limitations by proposing a comprehensive solution in which low quality factor (low-Q), omnidirectional polyvinylidene fluoride (PVDF) sensors are employed to replace conventional PZT transducers coupled with miniature microphones (MICs) to achieve simultaneous acquisition of forward and reverse wind signals, thereby simplifying the system structure. Regarding the algorithmic framework, variational mode decomposition (VMD) was first utilized to adaptively decompose non-stationary echo signals, effectively separating noise and suppressing mode mixing. Subsequently, a zero-wind speed reference signal was introduced to optimize the EKF observation model for envelope restoration of the reconstructed signal, and a hybrid approach combining cross-correlation and ZCD was adopted to enhance the accuracy of ToF acquisition. The proposed method significantly improved the accuracy and robustness of wind speed measurements under conditions of complex noise and low sampling rates.

## 2. Wind Speed Measurement Principle of PVDF Ultrasonic Sensors

**Figure 1:**
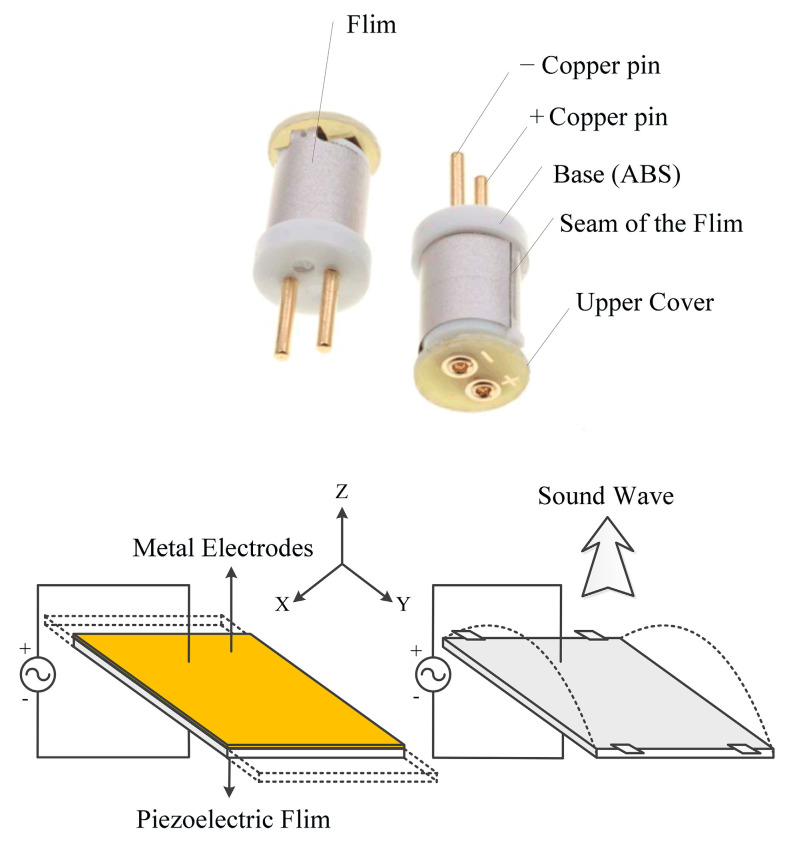
Schematic of the working principle of the polyvinylidene fluoride (PVDF) ultrasonic sensor.

PVDF, i.e., a typical flexible piezoelectric and pyroelectric polymer, exhibits low acoustic impedance, which enables good impedance matching with common acoustic transmission media such as air and water, thereby effectively improving sound energy transmission efficiency [16,17]. In this system, the 80 kHz cylindrical ultrasonic transducer utilizes a PVDF thin film with a thickness of only 30 μm. The operational resonance frequency ranges between 80 and 90 kHz, characterized by an exceptionally low quality factor (Q value); in particular, the Q value is only 4–8 in the transmission mode and 6–9 in the reception mode, with a typical overall system Q value of approximately 5.4. PVDF piezoelectric materials possess a low mechanical quality factor, indicating a high intrinsic damping that enables the rapid decay of residual vibrations, resulting in an extremely short pulse response time. Furthermore, its lightweight and flexible nature enables processing into films of various shapes with thicknesses ranging from tens of micrometers to millimeters. Benefiting from high damping and a low quality factor, PVDF films demonstrate a flat frequency response over a very broad frequency range, with shorter rise and decay times for acoustic signals compared with conventional piezoelectric ceramics [18]. These characteristics render PVDF particularly suitable for manufacturing high-resolution ultrasonic anemometers, enabling high-precision and rapid-response wind speed measurements through accurate ToF detection of ultrasonic waves. In this system, the piezoelectric film used in the cylindrical ultrasonic emitter operates in the d31 length extension mode when driven by low-frequency voltages (below 100 kHz). In this mode, if both ends of the piezoelectric film are fixed, length variations are induced that displace air to generate acoustic waves. Figure 1 depicts the operational mechanism [19].

**Figure 2:**
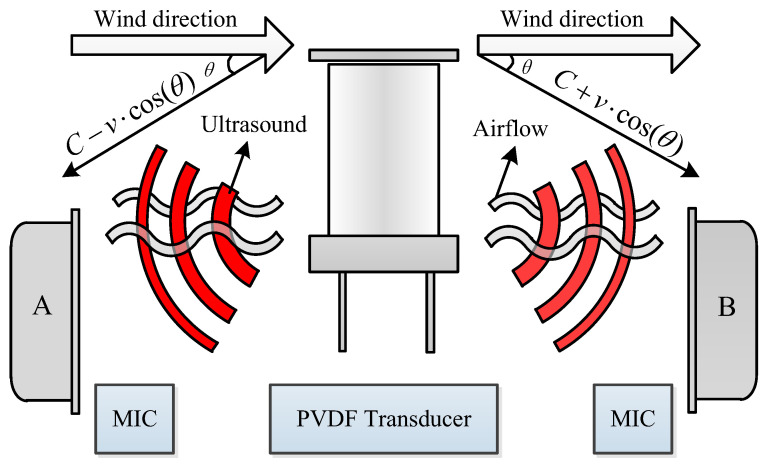
PVDF ultrasonic sensor measurement model.

Most conventional ultrasonic wind speed measurement systems require sequential driving of ultrasonic transducers, in which each unit functions as both a transmitter and a receiver. These systems utilize direct and inverse piezoelectric effects to transmit and receive ultrasonic signals propagating along or against the airflow direction and calculate wind speed based on the time difference between separate downstream and upstream measurements. Conversely, this study employs an 80 kHz omnidirectional PVDF ultrasonic transducer from TE Connectivity as the transmitter, while the receiving end utilizes two high-performance MEMS microphones (IMP23ABSU) from STMicroelectronics. The PVDF transducer features a horizontal beamwidth of 360° and a vertical beamwidth of ±25°. Consequently, when the wind direction was fixed, a single ultrasonic emission allowed MIC A to capture the upstream signal and MIC B to capture the downstream signal simultaneously. This configuration enables the synchronous acquisition of ToF variations induced by airflow. Figure 2 depicts the measurement principle.

In this system, the transducer functioned solely as a transmitter. Compared with conventional ultrasonic wind speed measurement systems, this approach simplifies the measurement process. Simultaneously capturing the upwind and downwind ultrasonic signals improved the measurement accuracy. The impact of the shadow effect was minimized by setting the horizontal angle θ between the receiving microphone and ultrasonic transducer to 25°. The wind speed *v* was calculated as follows:

(1) $$v=\frac{d}{2}\left(\frac{1}{\tau_{A}}-\frac{1}{\tau_{B}}\right),$$

where $\tau_{A}$ denotes the ToF from the transducer to MIC A, $\tau_{B}$ denotes the ToF from the transducer to MIC B, and *d* denotes the distance between the transmitter and receiver. An analysis of the measurement principle revealed that the time difference between the upwind and downwind signals was a critical parameter. Unlike ultrasonic liquid flowmeters, ultrasonic gas flowmeters are characterized by relatively low transmission and reception efficiencies. Consequently, the received ultrasonic signals are weak and susceptible to environmental noise interference, hindering the accurate extraction of time-difference information. Therefore, applying filtering and denoising techniques to the signals is essential.

## 3. Received Signal Processing Method

**Figure 3:**
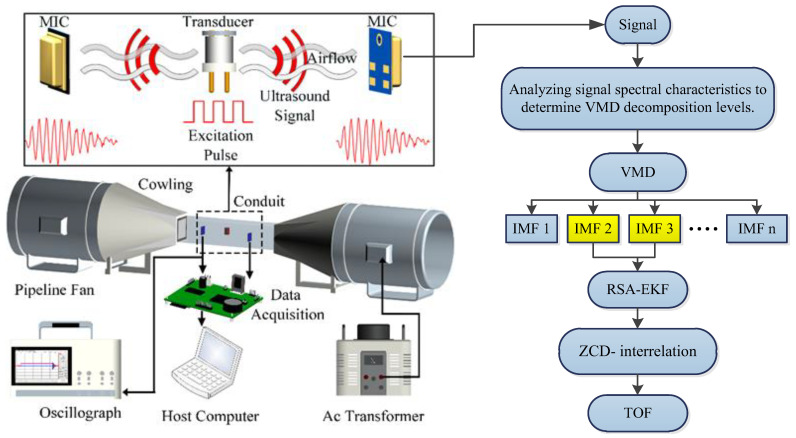
Framework diagram of the system and algorithm.

As the wind speed increases, the received ultrasonic signals are affected by turbulent disturbances and environmental noise. This study proposes an adaptive signal processing method based on the joint optimization of VMD and an EKF. First, VMD was used to separate noise from the signal. Effective IMFs were then selected for signal reconstruction. Subsequently, the EKF was applied to the reconstructed signal for envelope recovery, thereby improving the SNR of the received signal. The number of effective components, and thus the number of decomposition layers, was adaptively determined by analyzing the frequency-domain characteristics of the signal and setting a threshold. The signal was decomposed into several IMFs with distinct center frequencies. After selecting the effective modes, a preliminary reconstructed signal was obtained by summing the IMFs. The received signal at zero wind speed was recorded as a reference. The initial parameters of the EKF were optimized by combining the sensor characteristics and reference signal. The EKF was then used to filter the preliminarily reconstructed signal, repair its envelope, and obtain the final processed ultrasonic signal; a segment of the characteristic signal was extracted using the ZCD-interrelation method and cross-correlated with the reference signal to accurately determine the ToF. Figure 3 depicts a detailed flowchart of the algorithm.

### 3.1. Introduction to VMD

VMD is a non-recursive signal processing method based on the variational optimization theory [20,21]. The core philosophy of this approach involves formulating signal decomposition as a constrained optimization problem to identify a set of band-limited IMFs. Unlike the recursive sifting process of EMD [22], VMD employs a concurrent processing architecture that adaptively updates the center frequency and bandwidth of each mode in the frequency domain via a Wiener filtering mechanism. The specific decomposition steps of VMD are detailed in [23,24].

### 3.2. Choice of Penalty Factor α

The quadratic penalty factor *α* is a critical parameter determining the decomposition performance, as it directly controls the bandwidth constraint strength of the mode components. The mathematical objective of VMD is to minimize the sum of the estimated bandwidths of the center frequencies for all modes subject to an accurate reconstruction of the original signal by these components [25]. This constrained variational problem is solved by introducing a quadratic penalty factor *α* and a Lagrange multiplier λ to transform it into an unconstrained optimization problem. At this point, the augmented Lagrangian function L of the system is expressed as follows:

(2) $$L=\alpha \underset{k}{\sum }{\left\|\partial_{t}\left[\left(\delta +\frac{j}{\pi t}\right)*u_{k}\right]e^{-j\omega_{k}t}\right\|}_{2}^{2}+{\left\|f-\underset{k}{\sum }u_{k}\right\|}_{2}^{2}+\left\langle \lambda ,f-\underset{k}{\sum }u_{k}\right\rangle ,$$

where $u_{k}$ represents the *k*-th mode component, $\omega_{k}$ is the center frequency of each mode, δ denotes the Dirac delta function, and *f* is the original input signal.

The penalty factor, *α*, plays a critical role in the algorithm by balancing data fidelity with mode smoothness. When *α* is small, the bandwidth constraint is relaxed, allowing modes to encompass broader spectral components; although this enhances reconstruction accuracy, it may also introduce high-frequency noise interference. Conversely, a larger *α* strengthens the bandwidth constraint, forcing the modes to be more compact in the frequency domain (i.e., exhibiting narrow-band characteristics). This facilitates noise suppression; however, an excessively large value may lead to the loss of effective signal details or waveform over-smoothing. Fundamentally, VMD identifies the intrinsic oscillation patterns within the signal by modulating *α*. For actual physical signals with stable frequency characteristics, such as the ultrasonic echoes used in this study, VMD demonstrates excellent robustness. When *α* falls within a reasonable range, the algorithm prioritizes convergence to the true physical components of the signal. In this scenario, fine-tuning *α* primarily affects the transition band characteristics at the frequency boundaries (e.g., the side-lobe attenuation rate), with minimal impact on the −3 dB main-lobe bandwidth. Zhang et al. [26] investigated the denoising method for UHF partial discharge signals, noting that such signals possess physical characteristics similar to those of ultrasonic pulses, and recommended *α* = 2000 as a robust empirical value for balancing denoising effectiveness with feature preservation. A parametric sensitivity analysis was conducted using actual ultrasonic signals measured at a wind speed of 40 m/s to determine the optimal parameter applicable to the proposed system. With the decomposition level *K* fixed, VMD was performed with *α* set to 1000, 2000, and 5000; the resulting bandwidths of the IMFs are presented in Table 1.

The data analysis revealed that when *α* = 1000, the bandwidths of certain IMFs were excessively wide (>30 kHz), resulting in the erroneous inclusion of broadband noise. When *α* = 5000, the bandwidths were overly constrained (<10 kHz), leading to the loss of pulse envelope information and waveform distortion. By contrast, when *α* = 2000, the bandwidths of the IMFs converged within a reasonable range of 12–25 kHz. Consequently, *α* = 2000 was selected for processing the received signals, thereby achieving an optimal balance between SNR enhancement and waveform reconstruction accuracy.

### 3.3. Determination of VMD Decomposition Levels Based on Spectral Peak Detection

In the VMD algorithm, the number of modes *K* selected is of great importance. A *K* value that is too small results in under-decomposition, leading to mode mixing, whereas a *K* value that is too large causes over-decomposition, generating spurious modes or misidentifying noise as signal components [27]. Leveraging the principle that significant peaks in a signal spectrum typically correspond to oscillation components with a clear physical meaning, this study employs a fast Fourier transform (FFT) to detect spectral peaks in the received signal and determine an appropriate *K* value. This method estimates the optimal number of decomposition levels by counting the significant peaks in the amplitude spectrum that exceed the dynamic threshold. The specific steps taken are as follows:

1. Spectrum calculation and normalization: FFT was performed on the discretized ultrasonic received signal. Given the conjugate symmetry of the spectrum for real-valued signals, only the single-sided spectrum (ranging from 0 to the Nyquist frequency *fs*/2) was retained, followed by amplitude normalization.
2. Preliminary screening of extrema points: The amplitude spectrum sequence was traversed. For internal data points, if the amplitude was strictly greater than that of both the preceding and succeeding neighbors, the point was marked as a candidate local maximum. For the endpoints, the comparison was performed only against a single adjacent neighbor.
3. Dynamic threshold determination: The global maximum of the amplitude spectrum, *A*_max_, was calculated. A threshold coefficient *η* was introduced (set to an empirical value of *η* = 0.1 in this study) to construct an adaptive noise threshold defined as *T*_h_ = *η* × *A*_max_. This threshold was dynamically adjusted according to the energy level of the current signal, thereby accommodating environments with varying SNR.
4. The amplitudes of all candidate peaks identified in Step 2 were evaluated against the dynamic threshold *T*_h_. Spurious peaks with amplitudes lower than *T*_h_, typically induced by noise, were eliminated, retaining only the significant peaks with amplitudes greater than or equal to the threshold.
5. Determination of decomposition level *K*: The number of the finally screened significant peaks was counted and assigned as the preset number of decomposition modes, *K*, for the VMD algorithm. This facilitated the adaptive extraction of effective components from the ultrasonic signal.

### 3.4. Comparative Analysis of the Decomposition Capabilities of VMD and EMD

**Figure 4:**
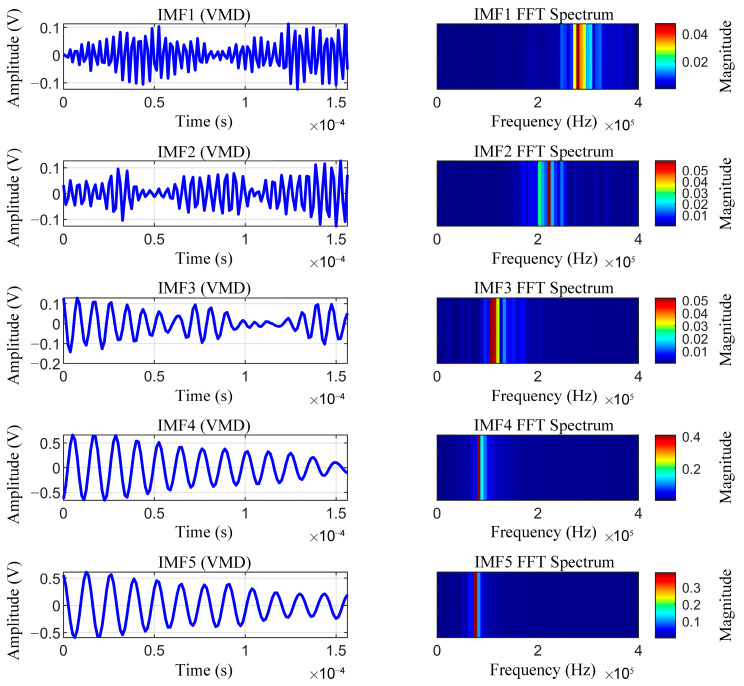
IMFs and frequency spectra obtained by variational mode decomposition (VMD).

**Figure 5:**
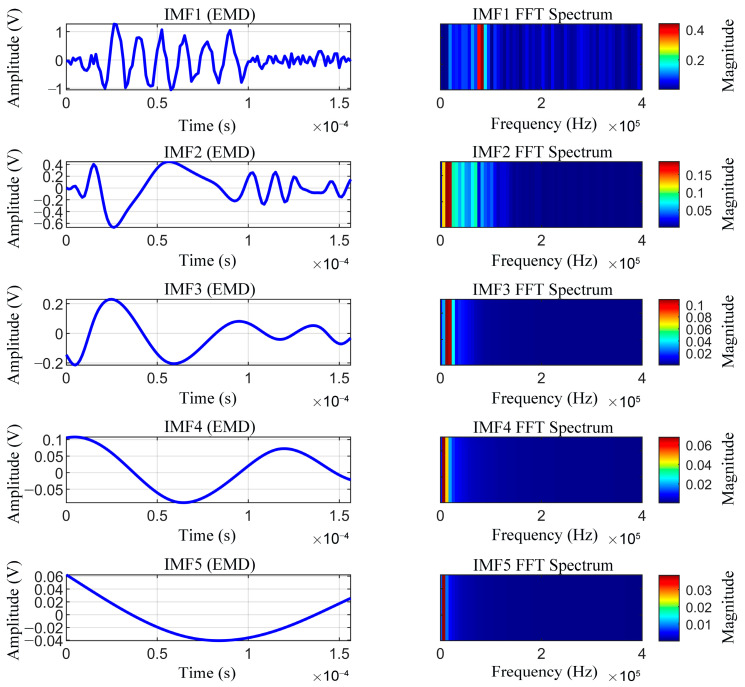
IMFs and frequency spectra obtained by empirical mode decomposition (EMD).

Influenced by factors such as wind speed and hardware noise, the instantaneous frequency of the received ultrasonic signal spans multiple frequency bands, leading to timescale aliasing within a single mode. The decomposition capability of VMD was demonstrated by employing both the VMD and EMD methods to decompose the same ultrasonic signal into five IMFs, and the spectra for IMF1–IMF5 were calculated. As illustrated in Figure 4 and Figure 5, VMD significantly outperformed EMD in terms of signal decomposition precision and resistance to mode mixing. In particular, the EMD results exhibit significant spectral overlap among multiple IMFs; particularly, in the high-frequency region, the oscillation components of varying timescales are commingled within a single IMF, which severely compromises the reliability of feature extraction. Conversely, VMD operates on the assumption that each mode is a narrow-band signal centered at a fixed frequency. By constructing a variational optimization problem, VMD transforms the signal decomposition into a constrained bandwidth minimization process, thereby achieving explicit control over the center frequency and bandwidth of each mode. The resulting IMFs exhibited highly concentrated narrow-band characteristics in the frequency domain, characterized by distinct spectral boundaries and minimal overlap, making this method more suitable for the decomposition of received ultrasonic signals.

Effective IMFs are selected by leveraging the decomposition capability of VMD and then superimposed based on whether their spectral center frequencies fall within the operating bandwidth of the transducer (78–92 kHz). This approach effectively suppresses uncorrelated noise components while preserving the primary features of the ultrasonic waveform, thereby yielding high-quality input data for subsequent signal processing.

### 3.5. Signal Envelope Restoration and Waveform Optimization Based on Reference Signal-Assisted EKF

Although the studies by Angrisani et al. and other researchers [28] verified the effectiveness of the EKF in ultrasonic ToF estimation, their experiments were mostly limited to controlled environments and failed to fully address the problems of waveform distortion and frequency shifts caused by turbulence in actual wind speed measurements. When processing signals with low SNR or strong distortion, conventional EKF algorithms can easily lead to the divergence of state estimation and the phenomenon of “model mismatch” if initial parameters are improperly set or an accurate observation model is lacking [29]. This study addresses these limitations by proposing an improved EKF algorithm based on a signal reconstructed by VMD screening. The core innovation of this algorithm is the introduction of a zero-wind-speed reference signal to construct a nonlinear observation model that constrains state estimation within the range of physically realistic waveform characteristics. By effectively separating a part of the noise through VMD preprocessing and combining it with the reference signal-assisted EKF for secondary filtering and envelope restoration of the reconstructed signal, the robustness of the algorithm under complex flow fields was significantly improved. The specific implementation steps are as follows:

1. Establishment of the state-space model: The amplitude attenuation coefficient and time delay of the ultrasonic signal during propagation are defined as the state variables of the system. The state vector is defined as [30]
  (3) $$x_{k}={\left[a_{k},\tau_{k}\right]}^{T},$$
  where $a_{k}$ denotes the signal amplitude scaling factor at time k, and $\tau_{k}$ denotes the time shift in units of the sampling points. The state covariance matrix ***P***_0_ and process noise covariance matrix ***Q*** are initialized as follows:
  (4) $$P_{0}=\left[\begin{array}{cc} \sigma_{a}^{2} & 0\\ 0 & \sigma_{\tau }^{2} \end{array}\right],\;Q=\left[\begin{array}{cc} q_{a} & 0\\ 0 & q_{\tau } \end{array}\right],$$
2. Construction of the nonlinear observation model: The issue of waveform distortion is addressed by introducing a standard ultrasonic signal *S*_ref_(*t*), previously acquired in a zero-wind-speed environment, as the basis for the observation model. The observation equation of the system at time *k* is modeled as the result of the reference signal modulated by the amplitude and time delay [31]
  (5) $$z_{k}=h\left(x_{k}\right)+v_{k}=a_{k}\cdot s_{ref}\left(t_{k}-\tau_{k}\right)+v_{k},$$
  where *z_k_* denotes the actual observed signal reconstructed via VMD, *v_k_* denotes the observation noise, and *t_k_* = *k*/*F_s_*, where *F_s_* denotes the sampling frequency. This model significantly strengthens the physical constraints imposed on the filtering process by directly correlating the estimated states with the reference signal.
3. Calculation of the Jacobian matrix (linearization). Because the observation equation $h\left(x_{k}\right)$ is nonlinear, the EKF algorithm involves linearizing it at each iteration by performing a Taylor series expansion, i.e., by computing the Jacobian matrix $H_{k}$.
  (6) $$H_{k}={\left.\frac{\partial h(x)}{\partial x}\right|}_{{\hat{x}}_{k|k-1}}=\left[S_{ref}\left(t_{k}-{\hat{\tau }}_{k|k-1}\right)-{\hat{\alpha }}_{k|k-1}\cdot S_{ref}^{\prime }\left(t_{k}-{\hat{\tau }}_{k|k-1}\right)\right],$$
  where *S’*_ref_ denotes the first-order derivative of the reference signal, computed via numerical differentiation. This derivative term incorporates information regarding the waveform rate of change, thereby endowing the filter with enhanced phase sensitivity at the waveform edges (high-frequency components). Given that *S*_ref_ is a discrete sampled sequence, its derivative is computed using the central difference method with a small perturbation step Δ*τ* as follows:
  (7) $$S_{ref}^{\prime }(t)\approx \frac{S_{ref}(t+\Delta \tau )-S_{ref}(t-\Delta \tau )}{2\Delta \tau },$$

This computation achieves the local linearization of the nonlinear system and is essential for performing the Kalman update.

4. EKF prediction and update iteration. The algorithm executes a standard predictor–corrector cycle, iteratively refining the state estimates to minimize the mean squared error (MSE) as follows:
  (8) $${\hat{x}}_{k|k-1}={\hat{x}}_{k-1|k-1},\;P_{k|k-1}=P_{k-1|k-1}+Q,$$

Calculate the Kalman gain **K***_k_*_._

(9) $$K_{k}=P_{k|k-1}H_{k}^{T}{\left(H_{k}P_{k|k-1}H_{k}^{T}+R\right)}^{-1},$$

Measure the residual ${\tilde{y}}_{k}$

(10) $${\tilde{y}}_{k}=z_{k}-h\left({\hat{x}}_{k|k-1}\right),$$

Update the state.

(11) $${\hat{x}}_{k|k}={\hat{x}}_{k|k-1}+K_{k}{\tilde{y}}_{k}.$$

5. Post-processing and physical constraints: Physical constraints are imposed on the time delay estimate $\tau_{k}$ to prevent it from diverging into a nonphysical interval owing to noise interference during the initial iterations.
  (12) $$\tau_{\min}\leq {\hat{\tau }}_{k}\leq \tau_{\max}$$

Based on the aforementioned process, the reference signal-assisted EKF (RSA-EKF) algorithm yields the optimal estimated states, ${\hat{\alpha }}_{opt}$ and ${\hat{\tau }}_{\mathrm{opt}}$. The final restored signal $\hat{z}(t)$ can be expressed as

(13) $$\hat{z}(t)={\hat{\alpha }}_{\mathrm{opt}}\cdot S_{\mathrm{ref}}\left(t-{\hat{\tau }}_{\mathrm{opt}}\right)\hat{z}(t)={\hat{\alpha }}_{\mathrm{opt}}\cdot S_{\mathrm{ref}}\left(t-{\hat{\tau }}_{\mathrm{opt}}\right).$$

**Figure 6:**
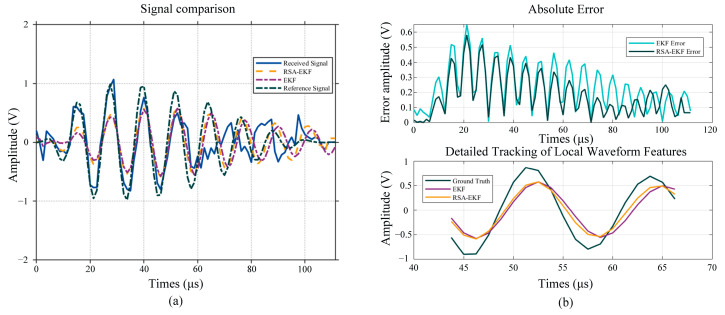
Performance comparison between reference signal-assisted extended Kalman filter (RSA-EKF) and standard EKF methods: (**a**) Global waveform reconstruction comparison; (**b**) absolute error analysis and detailed tracking of local waveform features.

Based on the analysis of the measurement principle, the time difference between the downstream and upstream propagation is a critical parameter. Unlike ultrasonic liquid flowmeters, ultrasonic gas flowmeters are characterized by relatively low transmission and reception efficiencies. Consequently, the received ultrasonic signals are weak and susceptible to environmental noise interference, hindering the accurate extraction of time-difference information. Therefore, applying filtering and denoising techniques to the signals is essential. Conventional EKF is vulnerable to frequency drift and phase lag under low-SNR conditions. Figure 6a depicts the filtering results of the reconstructed signal using both EKF and RSA-EKF. Quantitative analysis demonstrates that the root mean square error (RMSE) for the EKF is approximately 0.2828 V, whereas it decreases to 0.2301 V for the RSA-EKF. By introducing the standard transmission waveform as a prior frequency constraint, the RSA-EKF significantly reduces the absolute error relative to the reference signal, as shown in Figure 6b. Furthermore, the zoomed-in view in Figure 6b demonstrates that the RSA-EKF accurately tracks the phase variations. The noticeable instantaneous error amplitude is primarily attributed to physical waveform distortion at 40 m/s and algorithmic smoothing. For 80 kHz signals, even slight phase misalignments result in significant voltage differences. Crucially, this amplitude deviation does not compromise accuracy, as the combined cross-correlation and ZCD methods (Section 4) rely on zero-crossing points rather than absolute voltage.

**Figure 7:**
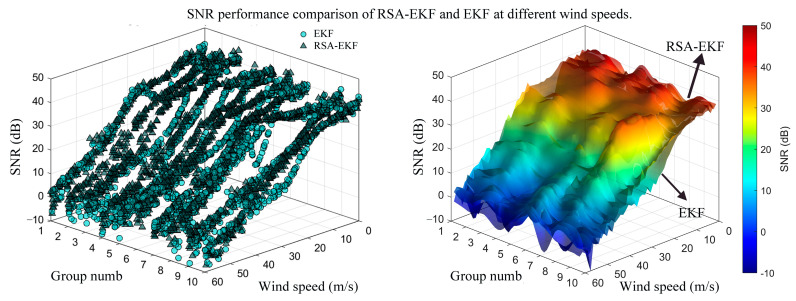
Signal quality enhancement via VMD-EKF: a comparative signal-to-noise ratio (SNR) analysis across wind speed groups.

Ten sets of ultrasonic received signals were collected under varying wind speed conditions to further demonstrate the performance of the RSA-EKF algorithm. The signals were reconstructed using VMD and denoising. Subsequently, both the EKF and RSA-EKF algorithms were applied to the processed data to calculate the SNR with respect to the standard reference signal. Figure 7 depicts the experimental results.

The statistical results indicated that the average SNR obtained after conventional EKF processing was 3.678 dB, whereas the average SNR of the signals processed by the joint RSA-EKF method increased to 4.657 dB, representing an improvement of approximately 26%. The SNR-fitted surfaces further elucidate the overall performance trends of the two methods when processing the received signals across varying wind speeds under identical experimental conditions. Notably, the surface area corresponding to the RSA-EKF was consistently higher than that obtained using the conventional EKF method. These results demonstrate that the proposed strategy, applying RSA-EKF filtering and envelope restoration following VMD decomposition and reconstruction preprocessing, can systematically enhance the signal quality.

## 4. Precise ToF Acquisition Method Based on ZCD Window Selection and Quadratic Cross-Correlation Interpolation

Owing to the measurement principle of the phase-difference method, the phase difference between the received signals cannot exceed half the period of the ultrasonic signal, which limits the wind speed measurement range [32]. A cascaded method for estimating the ToF is proposed to address this issue, which combines integer-cycle time delay resolution via cross-correlation with ZCD. This method first obtains the integer-cycle time delay by acquiring the cross-correlation peak between the received and reference signals through cross-correlation. Subsequently, it uses ZCD to obtain a precise sub-cycle phase deviation, thereby achieving high-precision measurements over a wide range [33]. The propagation time delay Δ*t* of the ultrasonic signal can be decomposed as follows:

(14) $$\Delta t=n\cdot T_{0}+\Delta t_{\phi },$$

where *n* is the number of full signal delay periods, *T*_0_ is the period of the ultrasonic wave (for the 80 kHz transducer used in this system, *T*_0_ = 12.5 μs), and Δ*t* is the slight phase deviation of less than one full period. This method comprises the following steps:

1. Global estimation of integer-cycle time delay: VMD was applied to the zero wind-speed reference signal *x*_ref_(*k*) and measured signal *x*_meas_(*k*) for denoising. Their discrete cross-correlation function *R*_rm_(*m*) was calculated, and the lag index *m*_peak_ corresponding to its global maximum was identified. The number of full-period delays *n* was then determined based on the system sampling frequency *f*s and signal frequency *f*_0_.
  (15) $$n=round\left(\frac{m_{\mathrm{peak}}}{f_{s}\cdot T_{0}}\right)$$

Taking the solution that falls within the interval [*t_k_*_0_, *t_k_*_0+1_], the precise zero-crossing time difference between the reference and measured signals was calculated, yielding the following sub-cycle phase deviation:

2. Local refinement estimation of sub-cycle phase. To eliminate the influence of the macro-level delay on the phase measurement, the signals were first aligned by shifting the reference signal *x*_ref_(*k*) backward in the time domain by *n* periods. The first positive zero-crossing index *k*_0_ was then determined. A set of five sampling points around *k*_0_ (including *k*_0_ itself) was selected to construct a local quadratic polynomial fitting model *P*(*t*):
  (16) $$P(t)=at^{2}+bt+c.$$

3. The coefficients *a*, *b*, and *c* were solved using the least-squares method: By setting *P*(*t*) *=* 0 and solving the real root of this equation within the sampling interval, the precise zero-crossing time *t*_zcd_ at the subsampling level was obtained as follows:
  (17) $$t_{zcd}=\frac{-b\pm \sqrt{b^{2}-4ac}}{2a}.$$

Taking the solution that falls within the interval [*t_k_*_0_, *t_k_*_0+1_], the precise zero-crossing time difference between the reference and measured signals was calculated, yielding the following sub-cycle phase deviation:

(18) $$\Delta t_{\phi }=t_{zcd\_\mathrm{meas}}-t_{zcd\_\mathrm{ref}}\Delta t_{\phi }=t_{zcd\_\mathrm{meas}}-t_{\mathrm{zcd}\_\mathrm{ref}}.$$

4. ToF acquisition: The integer-cycle delay and phase deviation were linearly superimposed to synthesize the final ToF.
  (19) $$\Delta t=n\times 12.5\;\mu s+\Delta t_{\phi }$$

## 5. Experimental Data Acquisition

**Figure 8:**
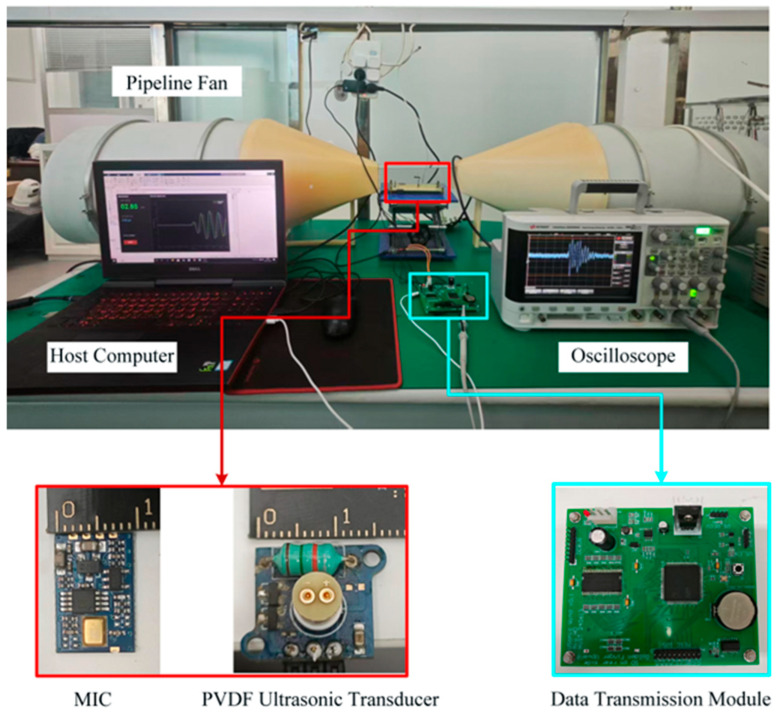
PVDF-based ultrasonic wind speed measurement system and wind-tunnel testing environment.

A dedicated high-speed airflow test platform was established to validate the reliability of the proposed method. The platform primarily comprised two axial fans, a flow straightener, an air duct, and a voltage regulator. A voltage regulator was coupled to both fans to simultaneously control the rotational speeds of the intake and exhaust units. As a large volume of air traversed the narrowed air duct, a stable airflow was generated within the channel. Figure 8 depicts the experimental setup for the wind speed testing environment.

**Figure 9:**
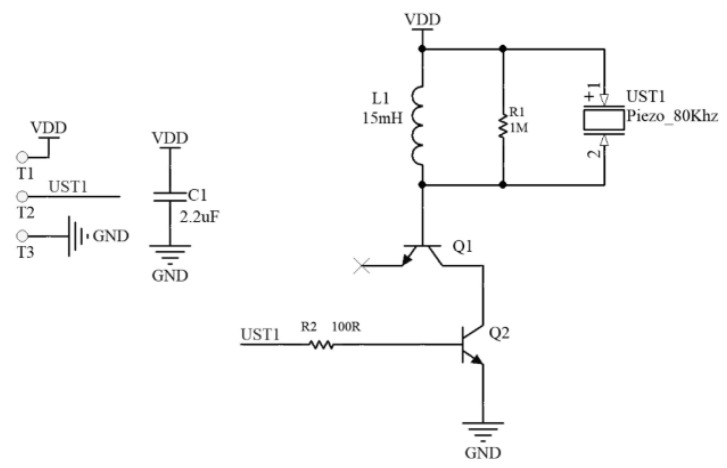
Schematic of the ultrasonic transducer drive circuit.

The core component of the transmitter was an omnidirectional PVDF piezoelectric film transducer (model: 80 kHz cylindrical ultrasound transducer). Owing to the relatively low piezoelectric constant of the PVDF material (d_31_ ≈ 20 pC/N) and its capacitive load characteristics (approximately 200 pF) under high-frequency driving, high-voltage excitation must be applied to obtain a sound pressure level (SPL) sufficient to overcome air attenuation. According to the principle of the inverse piezoelectric effect, the electroacoustic conversion efficiency is highest when the frequency of the alternating electric field matches the mechanical resonance frequency of the material. This system utilized a high-voltage drive circuit based on the LC resonance boost principle, as shown in Figure 9, to address the issues of the thick dielectric layer of the PVDF film and the need to overcome the acoustic impedance mismatch with air. The circuit employed a complementary push–pull structure (Q1/Q2) as the power stage, which worked in conjunction with a 15 mH high-Q inductor (L1) and the equivalent capacitance of the PVDF sensor to form a parallel resonant circuit. The 80 kHz PWM pulse signal generated by the MCU was amplified by the push–pull stage and then further boosted within the resonant circuit. Experimental tests indicated that this drive stage could generate a high-voltage sinusoidal excitation signal with a peak-to-peak value (V_pp_) greater than 130 V, effectively exciting the PVDF film to produce a high-SNR ultrasonic signal with a center frequency of 80 kHz.

## 6. Results and Discussion

**Figure 10:**
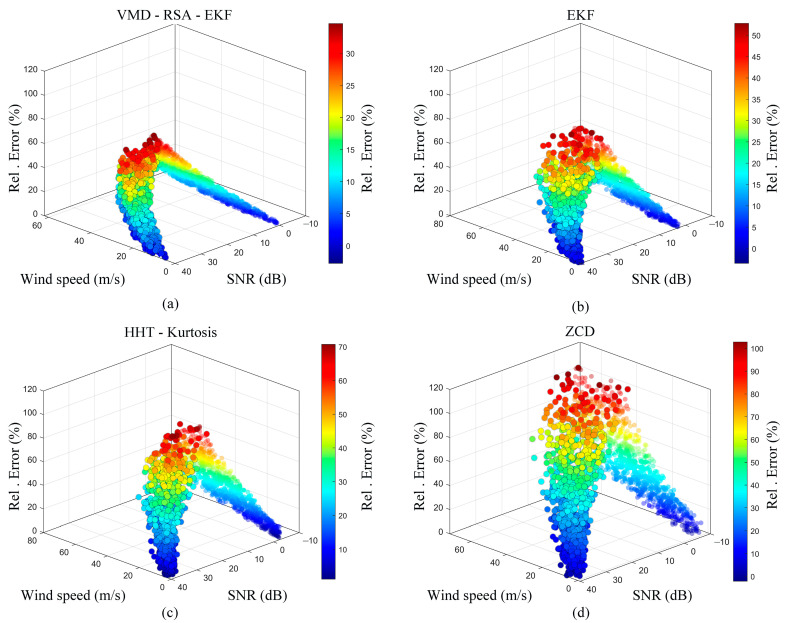
Comparison of the three-dimensional (3D) scatter plots of relative errors for four different algorithms under varying wind speeds and SNRs. (**a**) VMD-EKF-zero-crossing detection (ZCD, proposed method) showing distinct clustering in the low-error region and superior robustness; (**b**) Traditional EKF: exhibiting error diffusion at high wind speeds; (**c**) Hilbert–Huang transform (HHT)-Kurtosis: showing a divergent trend owing to mode mixing; (**d**) Traditional ZCD: displaying high dispersion and sensitivity to noise. The color bar indicates the magnitude of the relative error.

Systematic comparative experiments were conducted on an established wind speed test platform to validate the robustness and measurement accuracy of the proposed VMD-EKF-ZCD algorithm under varying flow velocity conditions. In total, 300 sets of ultrasonic echo signals were acquired across a range of wind speeds. The proposed VMD-EKF-ZCD algorithm was benchmarked against the conventional EKF filtering, ZCD, and the HHT–Kurtosis method. Figure 10 presents the three-dimensional (3D) scatter plots illustrating the distribution of the relative wind speed errors for the four methods under varying wind speed and SNR conditions.

Figure 10a depicts the experimental results of the VMD-EKF-ZCD algorithm. The scatter distribution exhibited significant clustering characteristics, indicative of a low error, with data points tightly aggregated within the low-error region (represented by the blue-to-green color bands). Even under severe operating conditions characterized by high wind speeds (>40 m/s) and low SNR (<10 dB), the relative error remained stable at a minimal level (generally below 40%), with no significant divergence observed. This indicates that by effectively isolating broadband noise via VMD frequency-domain decomposition and integrating it with the time-domain envelope restoration provided by the RSA-EKF, the proposed algorithm successfully mitigates the signal distortion induced by strong turbulence. The experimental results of the conventional EKF algorithm are shown in Figure 10b. Although this method performs satisfactorily in regions characterized by low wind speeds and high SNR, the scatter points rapidly diffuse toward high-error regions (indicated by yellow-to-red color bands) as the wind speed increases. This degradation is attributed to the conventional EKF’s lack of prior constraints regarding non-stationary distorted waveforms. Consequently, when the SNR deteriorates, the state estimation becomes highly susceptible to model mismatches, thereby triggering a rapid degradation in estimation accuracy. The experimental results of the HHT–Kurtosis algorithm are shown in Figure 10c. The error distribution exhibits a distinct divergent trend. Although the HHT possesses time–frequency analysis capabilities, the inherent limitations of EMD—specifically end effects and mode mixing—compounded by the sensitivity of the kurtosis metric to non-Gaussian noise, result in a significant degradation of measurement stability within high-wind-speed turbulent environments. Furthermore, relying solely on the ZCD method for ultrasonic ToF extraction yields substantial errors. As shown in Figure 10d, the scatter points exhibit high dispersion across the entire domain, with a significant number of data points falling into the high-error region (indicated by the red zone, corresponding to a relative error > 80%). This confirms that conventional time-domain threshold-based methods are highly sensitive to noise and sampling rate limitations, making them unsuitable for complex and dynamic wind-field environments.

The error composition is further clarified by decoupling the total measurement error (*E*_total_) into the algorithmic estimation error (*E*_algo_) and the physical fluctuation in the wind tunnel (*E*_env_) using the law of error propagation (*E*_total_^2^ = *E*_algo_^2^ + *E*_env_^2^). Under steady-state conditions, the wind tunnel maintains a velocity fluctuation (*E*_env_) within 1–5%. At the peak speed of 60 m/s, the wind tunnel contributes a maximum physical uncertainty of approximately 5% (3 m/s). With a total relative error of 32% observed at this speed, the intrinsic estimation error of the proposed VMD-EKF-ZCD algorithm is calculated to be approximately 34.6%. This quantification demonstrates that, despite the severe signal distortion and environmental turbulence at high wind speeds, the proposed algorithm effectively maintains a bounded and stable error range. This stands in sharp contrast to traditional methods like the ZCD, in which errors catastrophically diverge beyond 80% under identical conditions, thereby confirming the superior robustness and practical utility of the VMD-RSA-EKF framework in complex flow fields.

### Comparative Performance Analysis with Commercial Piezoelectric Ceramic Sensors

**Figure 11:**
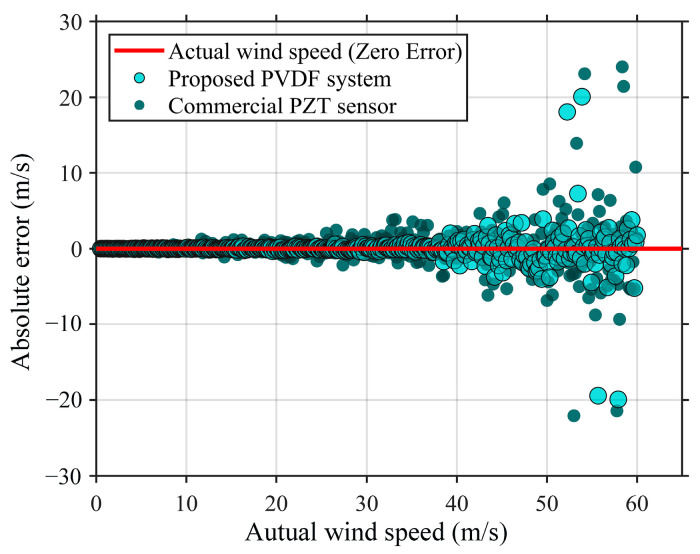
Comparison of the absolute measurement errors.

The practical utility of the proposed PVDF film-based ultrasonic wind speed measurement system was further validated by conducting a comprehensive comparative study using a commercial PZT ultrasonic wind sensing system. Figure 11 shows the absolute error distribution scatter plots for both systems across a wind speed range of 0–60 m/s. Between 0 and 40 m/s, the absolute error of the PVDF system demonstrates exceptional stability, with measurement points clustering tightly around the zero-error baseline represented by the red solid line. This indicates that the PVDF system exhibits superior linear response characteristics and minimal random dispersion throughout this initial measurement range.

As wind speed increases beyond 40 m/s, the measurement errors of both systems show a natural diverging trend due to environmental random disturbances such as turbulence. However, the error distribution of the PVDF system, represented by the teal dots, is significantly more convergent than that of the commercial PZT system. In particular, in the high-wind-speed region of 50–60 m/s, the PVDF system exhibits only a few outliers with absolute errors at approximately 20 m/s, whereas the overall error envelope remains relatively compact. By contrast, the commercial PZT sensor, shown as dark gray dots, exhibits more intense fluctuations in this interval, with several measurement errors exceeding the 20–25 m/s range, thus demonstrating significantly higher measurement uncertainty at high flow velocities.

The experimental results prove that the proposed wind speed measurement system based on a PVDF film demonstrates superior measurement linearity and environmental robustness across the entire range. By leveraging the wide bandwidth and low Q-factor characteristics of the PVDF, combined with the optimized VMD-RSA-EKF signal processing algorithm, the system is particularly effective under high-flow-velocity conditions where traditional commercial PZT systems tend to show increased divergence. This integrated approach from hardware mechanism to software algorithm forms a complete technical loop, providing an important theoretical basis and engineering reference for the development of precision meteorological monitoring instruments.

## 7. Conclusions

This study designed an ultrasonic sensing system based on a PVDF film, proposed a signal processing framework integrating VMD, and improved EKF and cross-correlation interpolation to address the critical demand for high-precision wind speed measurements in complex environments.

The experimental results demonstrate that the VMD-EKF-ZCD fusion algorithm exhibits superior robustness across the full wind speed range of 0–60 m/s and a wide SNR range of −10–40 dB. Through frequency-domain narrow-band filtering of VMD and time-domain envelope restoration of the RSA-EKF, this method effectively mitigates the non-stationary signal distortion induced by strong turbulence. Even under extreme conditions characterized by high wind speeds and low SNR, the system exhibits bounded error behavior, effectively constraining the relative error within a stable range (generally <50%), whereas conventional methods tend to diverge. The error distribution presents significant clustering–convergence characteristics, thereby proving its superior noise immunity and robustness.

In terms of hardware, comparative tests with commercial PZT sensors confirmed that the PVDF-based measurement system, leveraging its physical characteristics of wide bandwidth and low ringing, achieved higher measurement linearity and narrower error confidence intervals across the full range.

In summary, the scheme proposed in this study forms a complete technical closed loop, from a hardware mechanism to a software algorithm. This significantly enhances the accuracy and environmental adaptability of ultrasonic wind speed measurements and provides an essential theoretical basis and engineering reference for the development of precision instruments in fields such as meteorological monitoring and wind energy assessment.

In future works, this system can be upgraded to a three-dimensional (3D) wind speed and direction measurement system. The measurement of wind direction primarily depends on the decomposition of wind speed vectors; the flow direction is determined based on the wind speed components along various spatial directions. Therefore, the 3D wind information can be obtained by appropriately increasing the number of receiving microphones. This upgrade can enhance the omnidirectional perception capability of the system for complex flow fields, thereby enabling it to adapt to a wider range of meteorological monitoring requirements.

In terms of application prospects, the proposed system demonstrates significant potential for deployment in complex industrial environments. Attributed to the high sensitivity of the PVDF film and the robust noise immunity of the VMD-RSA-EKF algorithm, this system is particularly suitable for underground coal-mine ventilation monitoring. Moreover, its compact design and superior accuracy under low wind speed conditions render it an ideal candidate for portable meteorological stations integrated into unmanned aerial vehicles.

## Figures and Tables

**Table 1:** Impact of penalty factor *α* on the bandwidth of the decomposed intrinsic mode functions (IMFs).

α	IMF1	IMF2	IMF3	IMF4	IMF5
1000	15,882	14,797	28,657	28,856	34,087
2000	12,397	11,965	21,243	19,548	25,612
5000	8713	8526	20,086	13,234	18,842

## Data Availability

The data presented in this study are available on request from the corresponding author due to ongoing subsequent research on three-dimensional wind speed and direction.

## Abbreviations

The following abbreviations are used in this manuscript:

VMD	Variational mode decomposition
EKF	Extended Kalman filter
RSA-EKF	Reference signal-assisted extended Kalman filter
ZCD	Zero-crossing detection
HHT	Hilbert–Huang transform
EMD	Empirical mode decomposition
IMF	Intrinsic mode function
FFT	Fast Fourier transform
PVDF	Polyvinylidene fluoride
PZT	Piezoelectric ceramic transducer
MIC	Microphone
MEMS	Micro-electro-mechanical systems
MCU	Microcontroller unit
ToF	Time-of-flight
SNR	Signal-to-noise ratio
RMSE	Root mean square error
MSE	Mean squared error
SPL	Sound pressure level
PWM	Pulse width modulation
Vpp	Peak-to-peak voltage
High-Q	High-quality factor

## References

[1] Nosov V., Lukin V., Nosov E., Torgaev A., Bogushevich A. (2019). Measurement of Atmospheric Turbulence Characteristics by the Ultrasonic Anemometers and the Calibration Processes. Atmosphere.

[2] Sri Preethaa K.R., Muthuramalingam A., Natarajan Y., Wadhwa G., Ali A.A.Y. (2023). A Comprehensive Review on Machine Learning Techniques for Forecasting Wind Flow Pattern. Sustainability.

[3] Fang M., Xu K.-J., Zhu W.-J., Shen Z.-W. (2016). Energy transfer model and its applications of ultrasonic gas flow-meter under static and dynamic flow rates. Rev. Sci. Instrum..

[4] Ghaemi-Nasab M., Franchini S., Davari A.R., Sorribes-Palmer F. (2018). A procedure for calibrating the spinning ultrasonic wind sensors. Measurement.

[5] Shan Z., Lin G., Sun Z., Zhao Y., Xu K. (2023). Wind Speed and Direction Measurement Based on Three Mutually Transmitting Ultrasonic Sensors. IEEE Geosci. Remote Sens. Lett..

[6] Khyam M.O., Ge S.S., Li X., Pickering M.R. (2017). Highly accurate time-of-flight measurement technique based on phase-correlation for ultrasonic ranging. IEEE Sens. J..

[7] Liu T., Li Z., Zhang J., Li D., Dou H., Wu P., Yang J., Zhang W., Mu X. (2024). A Gas Flow Measurement System Based on Lead Zirconate Titanate Piezoelectric Micromachined Ultrasonic Transducer. Micromachines.

[8] Wang Z.-H., Liu X.-Y., Zhang H.-Q., Liu Y. (2022). Modeling of kinetic characteristics of alkaline-surfactant-polymer-strengthened foams decay under ultrasonic standing wave. Pet. Sci..

[9] Gómez Álvarez-Arenas T.E. (2013). Air-Coupled Piezoelectric Transducers with Active Polypropylene Foam Matching Layers. Sensors.

[10] Bao Y., Jia J. (2020). Improved time-of-flight estimation method for acoustic tomography system. IEEE Trans. Instrum. Meas..

[11] Herter S., Youssef S., Becker M.M., Fischer S.C.L. (2021). Machine Learning Based Preprocessing to Ensure Validity of Cross-Correlated Ultrasound Signals for Time-of-Flight Measurements. J. Nondestruct. Eval..

[12] Rincón M.J., Reclari M., Abkar M. (2022). Turbulent flow in small-diameter ultrasonic flow meters: A numerical and experimental study. Flow Meas. Instrum..

[13] Liu S., Tian Y., Wang G. (2021). Determining Ultrasound Arrival Time by HHT and Kurtosis in Wind Speed Measurement. Electronics.

[14] dos Santos J.C.F., Pinheiro P.P., de Franca J.A. (2019). Recovering of corrupted ultrasonic waves, for determination of TOF using the zero-crossing detection technique. IEEE Trans. Instrum. Meas..

[15] Sun M., Davies M.E., Proudler I.K., Hopgood J.R. (2023). Adaptive Kernel Kalman Filter. IEEE Trans. Signal Process..

[16] Mokhtari F., Cheng Z., Raad R., Xi J., Foroughi J. (2020). A Review on Wearable Electrospun Polymeric Piezoelectric Sensors and Energy Harvesters. Polymers.

[17] Zhu G., Zhou Y., Si Z., Cheng Y., Wu F., Wang H., Pan Y., Xie J., Li C., Chen A. (2023). A multi-hole resonator enhanced acoustic energy harvester for ultra-high electrical output and machine-learning-assisted intelligent voice sensing. Nano Energy.

[18] Fiorillo A.S. (2000). Noise analysis in air-coupled PVDF ultrasonic sensors. IEEE Trans. Ultrason. Ferroelectr. Freq. Control.

[19] Pullano S.A., Critello C.D., Bianco M.G., Menniti M., Fiorillo A.S. (2021). PVDF Ultrasonic Sensors for In-Air Applications: A Review. IEEE Trans. Ultrason. Ferroelectr. Freq. Control.

[20] Dragomiretskiy K., Zosso D. (2014). Variational mode decomposition. IEEE Trans. Signal Process..

[21] Rehman N.U., Aftab H. (2019). Multivariate Variational Mode Decomposition. IEEE Trans. Signal Process..

[22] Huang N.E., Shen Z., Long S.R., Wu M.C., Shih H.H., Zheng Q., Yen N.-C., Tung C.C., Liu H.H. (1998). The empirical mode decomposition and the Hilbert spectrum for nonlinear and non-stationary time series analysis. Proc. R. Soc. A Math. Phys. Eng. Sci..

[23] Zhang X., Miao Q., Zhang H., Wang L. (2018). A parameter-adaptive VMD method based on grasshopper optimization algorithm to analyze vibration signals from rotating machinery. Mech. Syst. Signal Process..

[24] Huang S., Sun H., Wang S., Qu K., Zhao W., Peng L. (2021). SSWT and VMD Linked Mode Identification and Time-of-Flight Extraction of Denoised SH Guided Waves. IEEE Sens. J..

[25] Wang Y., Markert R., Xiang J., Zheng W. (2015). Research on variational mode decomposition and its application in detecting rub-impact fault of the rotor system. Mech. Syst. Signal Process..

[26] Zhang J., He J., Long J., Yao M., Zhou W. (2019). A new denoising method for UHF PD signals using adaptive VMD and SSA-based shrinkage method. Sensors.

[27] Zhang M., Jiang Z., Feng K. (2017). Research on variational mode decomposition in rolling bearing fault diagnosis of the multistage centrifugal pump. Mech. Syst. Signal Process..

[28] Fürsattel P., Placht S., Balda M., Schaller C., Hofmann H., Maier A., Riess C. (2016). A Comparative Error Analysis of Current Time-of-Flight Sensors. IEEE Trans. Comput. Imaging.

[29] Reif K., Gunther S., Yaz E., Unbehauen R. (1999). Stochastic stability of the discrete-time extended Kalman filter. IEEE Trans. Autom. Control.

[30] Angrisani L., Baccigalupi A., Schiano Lo Moriello R. (2006). Ultrasonic Time-of-Flight Estimation Through Unscented Kalman Filter. IEEE Trans. Instrum. Meas..

[31] Khodarahmi M., Maihami V. (2023). A Review on Kalman Filter Models. Arch. Computat. Methods Eng..

[32] Jackson J.C., Summan V., Dobie G.I., Whiteley S.M., Hayward G., Pierce S.G. (2013). Time-of-Flight Measurement Techniques for Airborne Ultrasonic Ranging. IEEE Trans. Ultrason. Ferroelectr. Freq. Control.

[33] Jiang J.-J., Dang W., Duan F.-J., Wang X., Xiao F., Li C., Sun Z., Liu H., Bu L. (2020). An accurate ultrasonic wind speed and direction measuring method by combining time-difference and phase-difference measurement using coded pulses combination. Appl. Acoust..

